# A Novel Measure of Chromosome Instability Can Account for Prognostic Difference in Multiple Myeloma

**DOI:** 10.1371/journal.pone.0066361

**Published:** 2013-06-20

**Authors:** Tae-Hoon Chung, George Mulligan, Rafael Fonseca, Wee Joo Chng

**Affiliations:** 1 Cancer Science Institute of Singapore, National University of Singapore, Singapore, Singapore; 2 Millenium: The Takeda Oncology, Cambridge, Massachusetts, United States of America; 3 Mayo Clinic Comprehensive Cancer Center, Scottsdale, Arizona, United States of America; 4 Department of Medicine, Yong Loo Lin School of Medicine, National University of Singapore, Singapore, Singapore; 5 Department of Haematology-Oncology, National University Cancer Institute of Singapore, National University Health System, Singapore, Singapore; University of North Carolina at Chapel Hill, United States of America

## Abstract

Multiple myeloma (MM) is characterized by complex genetic abnormalities whose complexity signifies varying degree of chromosomal instability (CIN). In this study, we introduced a novel CIN measure, chromosome instability genome event count (CINGEC), which considered both copy number aberrations and interstitial breakpoints from high-resolution genome-wide assays. When assessed in two aCGH MM datasets, higher CINGEC was associated with poor survival. We then derived a CINGEC-associated gene expression profile (GEP) signature, CINGECS, using a dataset that has both aCGH and GEP. Genes in CINGECS were mainly involved in DNA damage responses besides in aneuploidy and other generic oncogenic processes contrary to other CIN associated GEP signatures. Finally, we confirmed its survival association in three GEP datasets that encompassed newly diagnosed patients treated with transplant-based protocol with or without novel agents for induction as well as relapsed patients treated with bortezomib. Furthermore, CINGECS was independent of many GEP-based prognostic signatures. In conclusion, our novel CIN measure has definite biological and clinical significance in myeloma.

## Introduction

Multiple myeloma (MM) is a plasma cell malignancy characterized by the accumulation of monoclonal plasma cell population in the bone marrow and pronounced chromosomal abnormalities. [1], [2] Almost all MM patients are characterized by genomic abnormalities including chromosome number and structural variations, although each case may differ significantly in the complexity of these abnormalities. The observed complexity is a clear indication of underlying genomic instability, the failure of protective cellular mechanism against the development of genomic abnormality and/or subsequent intrinsic oncogenic properties such as proliferation. The overall process that increases the rate of this genomic abnormality is conceptually captured as chromosome instability (CIN). [3] Although it is well established that individual abnormalities such as translocations (e.g. t(4;14) [4]) and deletions (e.g. 17p deletion [5]) or ploidy status (e.g. hypodiploid [6]) are associated with clinical outcomes, the true relevance of CIN in myeloma is unknown.

The detection of chromosome number and structural variations is used as a practical marker of CIN. In particular, recent developments of array-based high-resolution, high-throughput platforms such as array-based comparative genomic hybridization (aCGH) and single nucleotide polymorphism (SNP) chips have provided researchers with novel opportunities to investigate CIN in cancer genomes with resolutions that have never been possible with conventional assay techniques before [7]–[10].

In this study, we introduce a novel measure of CIN, chromosome instability genome event count (CINGEC), which incorporates structural alterations that are generally ignored in previous CIN indices that emphasized chromosome number variations and show that CINGEC is by itself a prognostic factor in myeloma. Subsequently, we develop a CINGEC-associated gene expression signature, CINGECS, from a public MM dataset that has both aCGH and gene expression profile (GEP) and assess biological mechanisms that are actively involved in the CIN phenomena by consulting two pathway repositories, Kyoto Encyclopedia of Genes and Genomes (KEGG) and gene ontology (GO). We also apply CINGECS to three public GEP datasets to examine its capacity to differentiate different prognostic groups either alone or in the presence of other GEP-based signatures and show that CINGECS is an independent prognostic factor in myeloma.

## Materials and Methods

### Chromosome Instability Genome Event Count (CINGEC)

CIN represents the tendency for a cell to be lenient towards compromises against genome integrity. Since a cancer cell with a more unstable genome will develop more aberration events, gain or loss of genome segments, until it experiences a systemic crisis, the degree of CIN of a genome can be assessed by counting the number of aberration events it harbors. In this study, we introduce CINGEC, the *heuristic minimum* number of aberration events inferred from genomic profiles, as a novel measure of CIN.

For the estimation of aberration events, we introduce two assumptions. First, we assume gains and losses are equally probable in all genome regions regardless of their copy number status. Second, we assume gains and losses of single or multiple copy numbers can happen with equal probability for a genome region. One consequence of these assumptions is that the chance of observing aberration events that happen at different time points sharing the same breakpoint is very slim. Another consequence is that all aberrant segments, irrespective of their spans and copy numbers, are equally important and should contribute as such to the estimation of CINGEC index.

**Figure 1:**
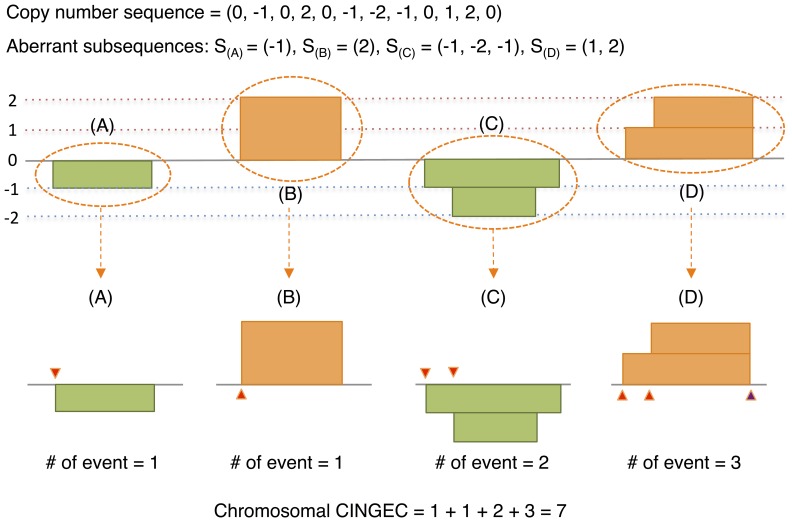
Schematic illustration of CINGEC algorithm for an artificial chromosome with a CNL sequence given by S = (0, -1, 0, 2, 0, -1, -2, -1, 0, 1, 2, 0). Aberrant subsequences for S are S_(A)_ = (-1), S_(B)_ = (2), S_(C)_ = (-1, -2, -1), S_(D)_ = (1, 2) and the number of events (marked by triangles in lower panels) for each subsequence following the algorithm is 1 for S_(A)_, 1 for S_(B)_, 2 for S_(C)_, 3 for S_(D)_ and the CINGEC for this chromosome is 7. Note that the event count for S_(B)_ is 1 as in S_(A)_ because CNL simply transits 0 → 2 in S_(B)_. Also note that the event count for S_(D)_ is 3 due to the last transition 2 → 0 (a transition into a level earlier than the immediate previous level 1) at the end of segment. In the algorithm, we assumed that two rugged end boundaries of levels 1 and 2 (as in S_(C)_) were truncated to an identical genomic locus by an additional event.

The CINGEC algorithm proceeds from a copy number sequence of a chromosome s = (s [1], …, s[n]), (s[i] ∈ {-p, …, q}; p, q >0; s[i] ≠ s[i+1]) obtained after discretizing aCGH data into copy number levels (CNLs) using segmentation (Figure 1). Here, positive and negative values represent different levels of gains and losses, respectively. Obviously, copy number sequence is composed of aberrant subsequences delimited by normal copy number segments (CNL = 0). In CINGEC, the number of aberration events of a chromosome is estimated by the sum of aberration events from aberrant subsequences. The number of aberration events of an aberrant subsequence increases by 1 if CNL transits into a new one (s[i] ∉ {s[j] (j<i)}) or CNL transits into earlier than the immediate previous level (s[i] = s[m], m<i-1). The latter criterion is based on the observation that the chance of two or more boundaries of independent aberration events coinciding with each other is very slim and it is more natural to assume an intervention of another aberration event that forces different breakpoints align with each other. If CNL returns to any of its previous levels, all intermediate CNLs between the departing and returning events will be expunged and estimation moves to next CNL. Final CINGEC estimate is the sum of all aberration events in autosomal chromosomes to avoid complications from sex chromosomes. Algorithmic details with an illustrative example are described in Method S1.

### Gene Expression Signature (CINGECS) Construction

Agilent 244K chip aCGH data of 254 MM patients from Multiple Myeloma Research Consortium (MMRC) reference collection were downloaded from Gene Expression Omnibus (GEO; GSE26849). [7] We segmented the aCGH data by using the CBS algorithm [11] implemented in ‘DNACopy’ R library [12] using default parameters and CINGEC values were estimated. MAS5 preprocessed Affymetrix HG-U133 Plus 2.0 GEP data for 304 MM patients from MMRC reference collection were downloaded from GEO (GSE26760). 246 of the MMRC samples had both aCGH and GEP data. We split CINGEC values of these samples into 4 quartiles and the differential gene expression between top and bottom quartile CIN groups was examined using the SAM algorithm [13] implemented in ‘siggenes’ R library [14]. Probesets with p-values ≤0.001 and false discovery rate (fdr) ≤0.05 and at least 2-fold expression difference between the top and bottom CIN groups were selected as CINGECS, the CINGEC-associated GEP signature.

### Pathway Analysis of CINGECS

In order to identify biological pathways enriched by member genes of CINGECS, we utilized impact factor (IF) analysis [15] implemented in Onto-Tools. [16] Contrary to many pathway-based analysis algorithms that consider only the enrichment of gene lists within specific pathways, IF analysis puts more emphasis on pathways whose member genes show fold changes that are congruent with underlying interaction topology. We also performed the GO analysis to supplement limited information obtained from IF’s KEGG-dependency.

### Survival Analyses Using Public aCGH and GEP Data

Prognostic utility of CINGEC and CINGECS was assessed through survival analysis using overall survival (OS). For CINGEC survival analysis, we used CINGEC scores estimated on aCGH datasets from the Mayo clinic (Mayo; Agilent 44K chip) [17] and from the University of Arkansas Medical School (UAMS; Agilent 22K chip) [18] separately. For CINGECS survival analyses, we used University of Arkansas dataset (UAMS; GSE2658; HG-U133 Plus 2.0) [19], [20] of 559 newly diagnosed MM patients treated with total therapy II & III, APEX clinical trial dataset (APEX; GSE9782; HG-U133 A/B) of 188 relapsed patients treated with bortezomib [21], and HOVON-65/GMMG-HD4 clinical trial dataset (HOVON; GSE19784; HG-U133 Plus 2.0) of 290 newly diagnosed MM patients [22], [23]. For APEX dataset, we used only HG-U133 A chip probesets in this study. Besides CINGECS, we used prognostic GEP signatures known to be statistically significant in MM and 2 previously reported CIN signatures that were predictive of patient prognosis in diverse cancers for comparison: 70-gene survival index developed by researchers from the University of Arkansas Medical School (UAMS70) [20], proliferation index (PI) [24], centrosome index (CNTI) [25], [26], 15-gene survival index from Intergroupe Francophone du Myelome study (IFM) [27], cell death genes affected by homozygous deletion (HZDCD) [28], 7-gene survival index from a detailed study of IL-6 dependent and independent MM cell lines (HMCL7) [29], 92-gene survival index from HOVON-65/GMMG-HD4 study (EMC92) [23], CIN index by Carter *et al*. (CIN70) [30] and CIN index from sarcoma study (CINSARC) [31]. (See Table S4 for full list of member probesets).

For each GEP dataset, we normalized the expression profile for a probeset by dividing individual expression values with the median across all samples. We, then, estimated the univariate CINGECS index of a sample by CINGECS = U – D where U is the logarithm (base 2) of median of normalized expression values of up-regulated CINGECS members while D is the logarithm (base 2) of median of normalized expression values over down-regulated CINGECS member genes, respectively. For other indices, the estimation was done as follows: indices UAMS70 and CNTI were estimated as indicated in their respective original publications. All other indices were estimated using log_2_-transformed median-normalized MAS5 signals as expression levels. For signatures where probesets are split into up- or down-regulation groups such as IFM and HZDCD, indices were estimated as done in CINGECS. All other indices were estimated as the median of expression levels of member probesets.

For each dataset, we performed univariate and multivariate survival analyses using Cox proportional hazard model. First, values from CINGEC index or GEP signature indices were respectively split into 4 quartile groups and survival tests using univariate Cox proportional hazard model were performed. For multivariate Cox analysis, we first examined three CIN-associated signatures (CINGECS, CIN70, CINSARC) to remove possible confounding effects due to similarity in signature construction and chose the best performing one (CINGECS) for further analysis. Multivariate Cox analyses were subsequently performed with remaining worsening GEP signatures from univariate analysis (HR >1 and p<0.05) and CINGECS. Stepwise refinements were applied at the end.

For data processing and analyses including survival analysis, we used R system [32] and its standard library ‘survival’. [33].

## Results

### CINGEC and Survival

We first estimated CINGEC scores of two MM aCGH datasets, 60 patient samples of the Mayo clinic and 100 patient samples of the UAMS collection, and compared them with the genome instability index (GII) that measures the fraction of aberrant genomic regions in a genome [34] (Figure S1). In both cases, CINGEC and GII were significantly correlated; correlation 0.62 (Figure S1(c); p = 4.668×10^−8^) for Mayo patient sample data and 0.43 (Figure S1(d); p = 9.194×10^−6^) for UAMS patient aCGH data. However, the data distribution suggests that aberration events covering whole chromosomes or arms make big impact on GII but little on CINGEC, whereas highly complicated copy number profiles with numerous small scale interstitial abnormalities clearly dominate samples with high CINGEC score (Figures S1 (c) and (d)).

**Figure 2:**
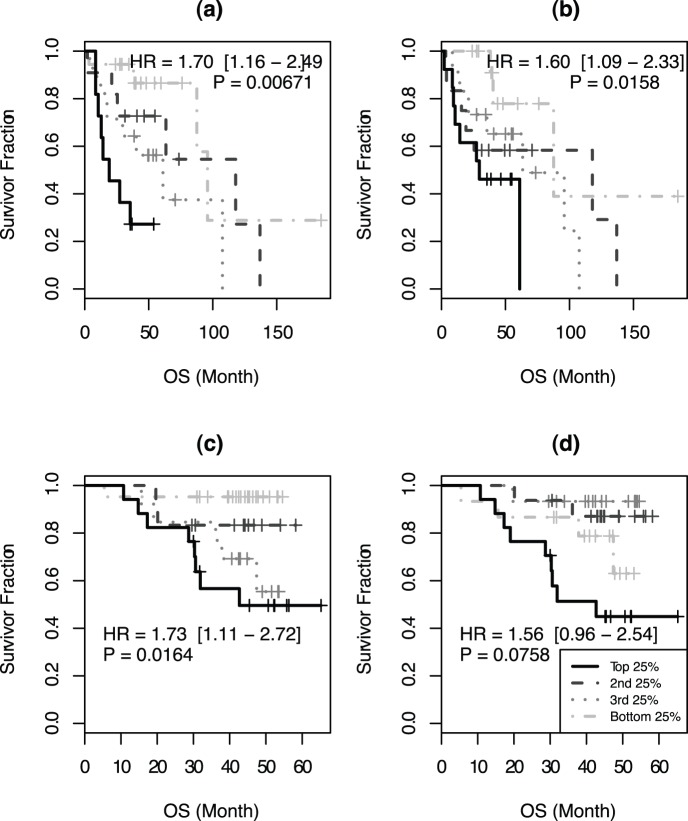
OS difference among different inter-quartile groups by (a) CINGEC, (b) GII of Mayo patient aCGH data and (c) CINGEC, (d) GII of UAMS patient.

Since CIN is known to cause adverse effects on patient survival in cancer, we tested if this was also the case in myeloma. Analysis using aCGH data from Mayo clinic clearly indicated that patients grouped according to their CINGEC score had significantly different OS (Figure 2(a); HR = 1.70 with 95% confidence interval (CI) = 1.16–2.49 and p-value = 0.00671). In contrast, the survival difference was not that significant when GII was used (Figure 2(b); HR = 1.60, CI = 1.09–2.33, p = 0.0158). In particular, the survival difference between the top quartile of CINGEC score and the rest quartiles combined (HR = 4.38, CI = 1.72–11.16, p = 0.00197) were substantially greater than in GII (HR = 2.74, CI = 1.12–6.74, p = 0.0281).

We next validated if this effect of CINGEC on prognosis was reproducible in an independent MM aCGH dataset. In the UAMS aCGH dataset where patients were treated on the total therapy II protocol, patients grouped according to their CINGEC score also had significantly different OS (Figure 2(c); HR = 1.73, CI = 1.11–2.72, p = 0.0164) while GII-based patient groups were not (Figure 2(d); HR = 1.56, CI = 0.96–2.54, p = 0.0758).

### CINGECS Genes and Pathways

To further understand the molecular difference between MM patients with high and low degrees of CIN, we analyzed the MMRC reference collection using data from 246 samples where both aCGH and GEP data were available. 214 probesets (160 genes; Table S1) were differentially expressed between samples in top 25% and bottom 25% CINGEC. 189 probesets (144 genes) were up-regulated and 25 probesets (16 genes) were down-regulated in the high CINGEC group.

As expected, many genes implicated in aneuploidy and DNA damage response were over-expressed in high CIN samples. Key regulators of cell cycle checkpoints, in particular those involved in the G2/M checkpoint (CDK1, CCNA2, CCNB1, CCNB2) and the mitotic checkpoints (AURKA, BUB1, BUB1B, CENPA, MAD2L1, NDC80, NEK2, PTTG1, TTK), were clearly over-expressed in high CIN samples. E2F, CDC gene families are well known cell cycle genes, and BIRC5, CENPA/F/H/K/N, KIF gene family, ZWINT are known to code proteins involved in kinetochore and microtubule attachment. On top of this, genes involved in mismatch repair pathway (EXO1, MSH2, PCNA, POLE2, RFC3/4/5), homologous recombination pathway (BRCA1, RAD51AP1), DNA damage signaling (CHEK1, RRM2, CCNB1/2, CDK1), and Fanconi anemia pathway (FANCI, UBE2T) were also over-expressed in high CIN samples. Furthermore, many genes in cancer-related pathways were also over-expressed in high CIN samples including proliferation (ASPM, CKS1B, MCM gene family, TOP2A, TTK, TYMS) and cancer testis antigens (MAGE family).

To make the observations from the list of CIN signature genes more concrete, pathways that were implicated by differentially expressed genes in high CIN MM were assessed by using the IF analysis first and then further complemented with the GO analysis (Table 1 and Figure S2). As expected, pathways implicated in aneuploidy (cell cycle and DNA replication) and DNA damage response (mismatch repair, nucleotide excision repair, p53 signaling pathway) were significantly enriched in the high CIN group. The results of GO analysis further consolidated the IF analysis results. The list of statistically significant biological process GO terms (Table S2) contained numerous cell cycle related terms (cell cycle (GO:0007049), cell division (GO:0051301), spindle organization (GO:0007051), mitosis (GO:0007067) etc.), DNA damage response terms (response to DNA damage stimulus (GO:0006974), DNA repair (GO:0006281), nucleotide-excision repair, DNA gap filling (GO:0006297) etc.), and oncogenic process terms (DNA replication (GO:0006260), cell proliferation (GO:0008283) etc.). CINGECS therefore appears to describe the CIN phenotype quite comprehensively. These functional associations of member genes also explain overwhelming dominance of up-regulated genes in high CIN samples in CINGECS.

**Table 1:** List of statistically significant KEGG pathways from IF analysis using CINGECS genes.

Pathway name	Impactfactor	Genes in pathway (number)	Input genes in pathway (number)	Pathway genes on chip (number)	Input genes in pathway (%)	Pathway genes in input (%)	Gamma p-value	Gamma p-value (corrected)	Enrichment p-value	Enrichment p-value (corrected)
Hsa04110: Cell cycle	29.424	118	16	112	10	13.6	5.06×10^−12^	5.06×10^−12^	5.20×10^−13^	5.20×10^−13^
Hsa03030: DNA replication	28.827	36	10	35	6.25	27.8	9.02×10−^12^	9.02×10^−12^	6.73×10^−13^	6.73×10^−13^
Hsa03430: Mismatch repair	18.631	23	6	22	3.75	26.1	1.59×10^−7^	1.59×10^−7^	1.83×10^−8^	1.83×10^−8^
Hsa03420: Nucleotide excision repair	11.355	44	5	43	3.13	11.4	1.45×10^−4^	1.45×10^−4^	2.58×10−^5^	2.58×10^−5^
Hsa04115: p53 signaling pathway	9.802	69	5	68	3.13	7.2	5.98×10^−4^	5.98×10^−4^	2.37×10^−4^	2.37×10^−4^

### CINGECS and Disease Prognosis

**Figure 3:**
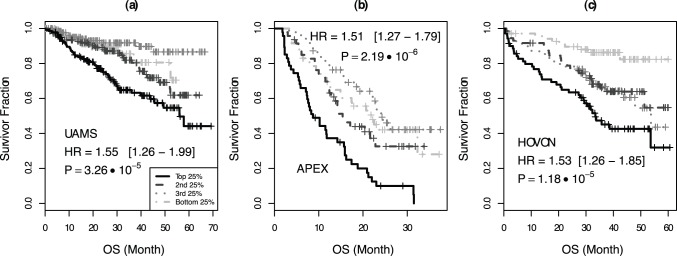
OS difference among different risk groups by CINGECS. (a) UAMS, (b) APEX, (c) HOVON dataset.

In order to assess the clinical relevance of CINGECS, we examined the association between CINGECS and OS using multiple public MM datasets. OS among CINGECS inter-quartile risk groups was statistically different in UAMS dataset (Figure 3(a); HR = 1.55, CI = 1.26–1.99, p = 3.26×10^−5^), in APEX dataset (Figure 3(b); HR = 1.51, CI = 1.27–1.79, p = 2.1×10^−6^), and in HOVON dataset (Figure 3(c); HR = 1.53, CI = 1.26–1.85, p = 1.18×10^−5^), respectively. In terms of clinical characteristics, there was no significant segregation of TC class across the CINGECS risk groups except for significantly more 11q13 cases in the high-risk CINGECS group in UAMS dataset (Table S3). Strikingly, CKS1B amplification was significantly more common in the high risk CIN group in UAMS dataset (65.1% in the highest CINGECS risk group compared to 28.1% in the lowest CINGECS risk group).

The prognostic utility of CINGECS in the presence of other GEP-based prognostic signatures was assessed by multivariate Cox proportional hazard analysis. First, to minimize the confounding effect, we compared CINGECS with two other CIN-associated signatures, CIN70 and CINSARC, that share substantial proportion of their member probes with CINGECS (33 and 42 probes out of 70 and 67 total probes, respectively; Figure S3) in three representative public datasets of MM (table 2). CINGECS consistently performed the best among CIN signatures in all datasets and retained for further multivariate analysis with various other prognostic GEP signatures of MM.

**Table 2:** Multivariate comparison of CIN-associated GEP signatures.

Dataset	Signature	HR (CI)	P
UAMS	**CINGEC**	**1.51 (1.20–1.91)**	**0.000483**
	CIN70	0.92 (0.60–1.40)	0.697
	CINSARC	1.15 (0.75–1.76)	0.530
APEX	**CINGEC**	**1.56 (1.28–1.89)**	**6.29×10^−6^**
	CIN70	1.04 (0.65–1.67)	0.869
	CINSARC	0.82 (0.52–1.31)	0.409
Hovon	**CINGEC**	**1.31 (1.06–1.61)**	**0.0127**
	CIN70	0.77 (0.43–1.35)	0.361
	**CINSARC**	**1.87 (1.06–3.30)**	**0.0308**

HR = Hazard Ratio; CI = 95% Confidence Interval; P = *p*-value.

In UAMS dataset, all signatures considered in this study were statistically significant for OS in univariate analyses (Table 3; individual survival curves in Figure S4) with CINGECS inferior only to UAMS70 (HR = 1.74, CI = 1.43–2.11, p = 3.33×10^−8^) and EMC92 (HR = 1.54, CI = 1.28–1.87, p = 7.4×10^−6^). On multivariate analysis, however, CINGECS (HR = 1.33, CI = 1.07–1.66, p = 0.0119) remained as an independent risk factor besides UAMS70 (HR = 1.54, CI = 1.25–1.90, p = 5.91×10^−5^) and HMCL7 (HR = 1.20, CI = 1.01–1.44, p = 0.0428). For OS in APEX dataset, all signatures except PI were statistically significant with CINGECS inferior only to EMC92 (HR = 1.53, CI = 1.30–1.82, p = 6.40×10^−7^) in univariate analyses (Table 3; individual survival curves in Figure S5). However, only CINGECS (HR = 1.38, CI = 1.16–1.64, p = 0.000258) and EMC92 (HR = 1.43, CI = 1.20–1.71, p = 5.87×10^−5^) were statistically significant on multivariate analysis. For OS in HOVON dataset, all signatures except HMCL7 were statistically significant in univariate analyses with EMC92 (HR = 2.27, CI = 1.85–2.80, p = 7.99×10^−15^), UAMS70 (HR = 1.66, CI = 1.37–2.02, p = 2.26×10^−7^), and PI (HR = 1.62, CI = 1.34–1.97, p = 1.02×10^−6^) superior to CINGECS (Table 3; individual survival curves in Figure S6). However on multivariate analysis, only EMC92 (HR = 2.14, CI = 1.73–2.65, p = 3.29×10^−12^) remained statistically significant beside CINGECS (HR = 1.26, CI = 1.04–1.53, p = 0.0198). Therefore, CINGECS was associated with poor outcome independent of other GEP signatures in both newly diagnosed and relapsed myeloma treated by either transplant-based therapy or novel agents.

**Table 3:** Summary of univariate and multivariate Cox proportional hazard analysis with stepwise refinement using various prognostic GEP signatures for OS in UAMS, APEX, and HOVON datasets.

		Univariate		Multivariate	
Data	Signature	HR (CI)	P	HR (CI)	P
UAMS	**CINGECS**	**1.55 (1.26–1.99)**	**3.26×10^−5^**	**1.33 (1.07–1.66)**	**0.0119**
	PI	1.24 (1.04–1.49)	0.0189		
	CNTI	1.30 (1.08–1.56)	0.00483		
	**UAMS70**	**1.74 (1.43–2.11)**	**3.33×10^−8^**	**1.54 (1.25–1.90)**	**5.91×10^−5^**
	IFM	1.42 (1.18–1.72)	0.000244		
	**HMCL7**	**1.22 (1.02–1.45)**	**0.0319**	**1.20 (1.01–1.44)**	**0.0428**
	HZDCD	1.37 (1.14–1.65)	0.000781		
	EMC92	1.54 (1.28–1.87)	7.4×10^−6^		
	**Overall**				**3.18×10^−9^**
APEX	**CINGECS**	**1.51 (1.27–1.79)**	**2.19×0^−6^**	**1.38 (1.16–1.64)**	**0.000258**
	PI	0.97 (0.82–1.14)	0.686		
	CNTI	1.25 (1.07–1.47)	0.00537		
	UAMS70	1.42 (1.20–1.68)	6.29×10^−5^		
	IFM	1.28 (1.09–1.51)	0.00209		
	HMCL7	0.84 (0.71–0.98)	0.0268		
	HZDCD	1.30 (1.10–1.54)	0.00215		
	**EMC92**	**1.53 (1.30–1.82)**	**6.40×10^−7^**	**1.43 (1.20–1.71)**	**5.87×10^−5^**
	**Overall**				**2.71×10^−9^**
Hovon	**CINGECS**	**1.53 (1.26–1.85)**	**1.18×10^−5^**	**1.26 (1.04–1.53)**	**0.0198**
	PI	1.62 (1.34–1.97)	1.02×10^−6^		
	CNTI	1.30 (1.08–1.56)	0.00467		
	UAMS70	1.66 (1.37–2.02)	2.26×10^−7^		
	IFM	1.53 (1.26–1.85)	1.27×10^−5^		
	HMCL7	1.20 (1.00–1.43)	0.0507		
	HZDCD	1.29 (1.07–1.55)	0.00660		
	**EMC92**	**2.27 (1.85–2.80)**	**7.99×10^−15^**	**2.14 (1.73–2.65)**	**3.29×10^−12^**
	**Overall**				**0**

HR = Hazard Ratio; CI = 95% Confidence Interval; P = *p*-value; Overall = collective p-value of final multivariate analysis with step-wise refinement.

## Discussion

Cancer genomes contain genomic alterations of variable complexity and CIN has been coined to characterize these compromised genomes. The existence of CIN in cancer genome clearly demonstrates malfunction or incompleteness of mechanisms responsible for maintaining genome integrity and detailed elucidation of molecular phenotypes associated with it can be very useful in understanding the etiology of cancer as well as clinical decision making since CIN has been shown to be associated with disease progression and chemotherapeutic responses.[35]–[38].

MM is characterized by highly complex genomic alterations. While specific genetic abnormalities have been associated with disease outcome [39], [40], the prognostic and biological relevance of underlying genome instability/complexity has not been well characterized in MM yet. Although we and others [26], [27] have reported high-risk signatures associated with expression of genes involved in mitotic checkpoints and postulated that dysregulation of genes involved in maintaining chromosomal integrity may indicate underlying CIN as an important mediator of poor prognosis, these were at best indirect inference as none of these studies have shown an association between expression of these signatures with a measure of CIN. A measure of CIN, CINGEC, estimated by a novel algorithm described in this study that assesses the number of aberration events necessary to account for both aneuploidy and structural alterations captured in high-throughput copy number data is shown to have direct association with disease progression and survival in MM. In addition, its associated GEP signature CINGECS was also significantly associated with poor prognosis in three independent MM datasets. Furthermore, it was independent of other prognostic signatures. Our results therefore strongly implicate CIN as a biologically and prognostically important factor in MM.

Comparison of CINGECS with two existing CIN signatures is quite illuminating. Carter *et al*. introduced a signature (CIN70) called total functional aneuploidy which is the sum of all absolute t statistic between expression levels of genes in a cytoband against average expression over the whole genome. [30] Recently, Chibon *et al*. derived a signature from sarcoma data (CINSARC) by combining aCGH imbalance comparison, histologic grade comparison, and CIN70 signature contents. [31] Since both CIN70 and CINSARC signatures are in principle based on aneuploidy and cell cycle, genes related to mitosis and proliferation are enriched in the signatures. However, CINGECS contains many DNA damage response related genes such as response to DNA damage stimulus, DNA repair, nucleotide-excision repair, DNA gap filling in addition to those enriched in aneuploidy centered signatures. This is a distinctive advantage of CINGEC estimation algorithm that considers all structural alterations equally regardless of genomic regions they span. It is also consistent with recent findings that defects in the response to DNA double-strand breaks [41] or in the homologous recombination [42] were implicated in mediating CIN and disease progression. Despite overlaps between genes constituting these CIN signatures, CINGECS but not CIN70 or CINSARC was consistently an independent prognostic factor in different datasets of MM. This suggests that aneuploidy only accounts for part of the prognostic impact of CIN in myeloma and the CINGECS more comprehensively describes the clinical relevance of CIN in MM.

CINGECS risk groups are not associated with biological features that endow preferential prognostic benefits or risks to TC classes such as 4p16 and MAF classes with bad prognosis [19] or D1 class with good prognosis. This suggests that CIN is independent of primary genetic events in myeloma and is probably driven by secondary mechanisms. However, the proportion of samples that have amplified CKS1B gene, another known risk factor [43], shows dramatic increase with CIN severity. This association suggests that CKS1B amplification is a late genetic event and a possible surrogate marker for advanced CIN. [44].

Finally, the prognostic impact of CINGECS is independent of other prognostic GEP signatures in MM. In fact, it remained independent in all datasets tested. This suggests that CIN potentially confers a unique aspect of poor outcome that is not captured in current landscape of prognostic signatures. This also suggests that different GEP signatures may exert different effect on myeloma outcome. As a consequence, one of the most important challenges in the future would be to understand the relationship between these independent signatures and whether they can be combined for better prognostic utility.

## Supporting Information

Figure S1 Genome wide segmentation heatmaps and scatter plots of CINGEC vs GII. Genome wide segmentation heatmaps of (a) Mayo patient primary sample aCGH data and (b) MMRC reference collection aCGH data, both in 1MB resolution. Samples are ordered according to increasing orders of CINGEC (middle panels). GII scores are displayed (bottom panels) for reference. Scatter plots between CINGEC vs GII for (c) Mayo patient data and (d) MMRC reference collection data are also shown. In heatmaps, single and 2+ copy number gains are shown in red and dark red, respectively. Likewise, single and 2 copy number losses are shown in greed and dark green, respectively.
(PDF)

Figure S2 KEGG pathway diagrams for pathways proved to be statistically significant from impact factor analysis (p<0.05); (a) cell cycle (hsa04110), (b) DNA replication (hsa03030), (c) mismatch repair (hsa03430), (d) nucleotide excision repair (hsa03420), (e) p53 signaling pathway (hsa04115). Over-expressed genes are indicated as red boxes in all diagrams.
(PDF)

Figure S3 Venn diagram of intersections among probesets in 3 CIN-related gene signatures.
(PDF)

Figure S4 Univariate survival curves for OS in UAMS dataset among inter-quartile risk groups of gene signature indices.
(PDF)

Figure S5 Univariate survival curves for OS in APEX dataset (bortezomib treatment cohort) among inter-quartile risk groups of gene signature indices.
(PDF)

Figure S6 Univariate survival curves for OS in HOVON dataset among inter-quartile risk groups of gene signature indices.
(PDF)

Table S1 CINGECS probeset list.
(XLS)

Table S2 Enrichment analysis results for GO terms using CINGECS genes. (a) Biological process terms. (b) Molecular function terms. (c) Cellular component terms.
(XLS)

Table S3 Association between clinical characteristics and CIN risk groups. (a) TC classification vs CIN risk groups for UAMS dataset. (b) CKS1B gain status vs CIN risk groups for UAMS dataset. (c) TC classification vs CIN risk groups for APEX bortezomib treatment dataset.
(XLS)

Table S4 List of probesets for MM prognostic signatures considered in this study.
(XLS)

Method S1 Supplementary Method.
(DOC)
