# Supplementary figures and images for: A Novel Measure of Chromosome Instability Can Account for Prognostic Difference in Multiple Myeloma
Source: PLoS One. 2013 Jun 20;8(6):e66361. doi: 10.1371/journal.pone.0066361 (PMC3688789; doi:10.1371/journal.pone.0066361)

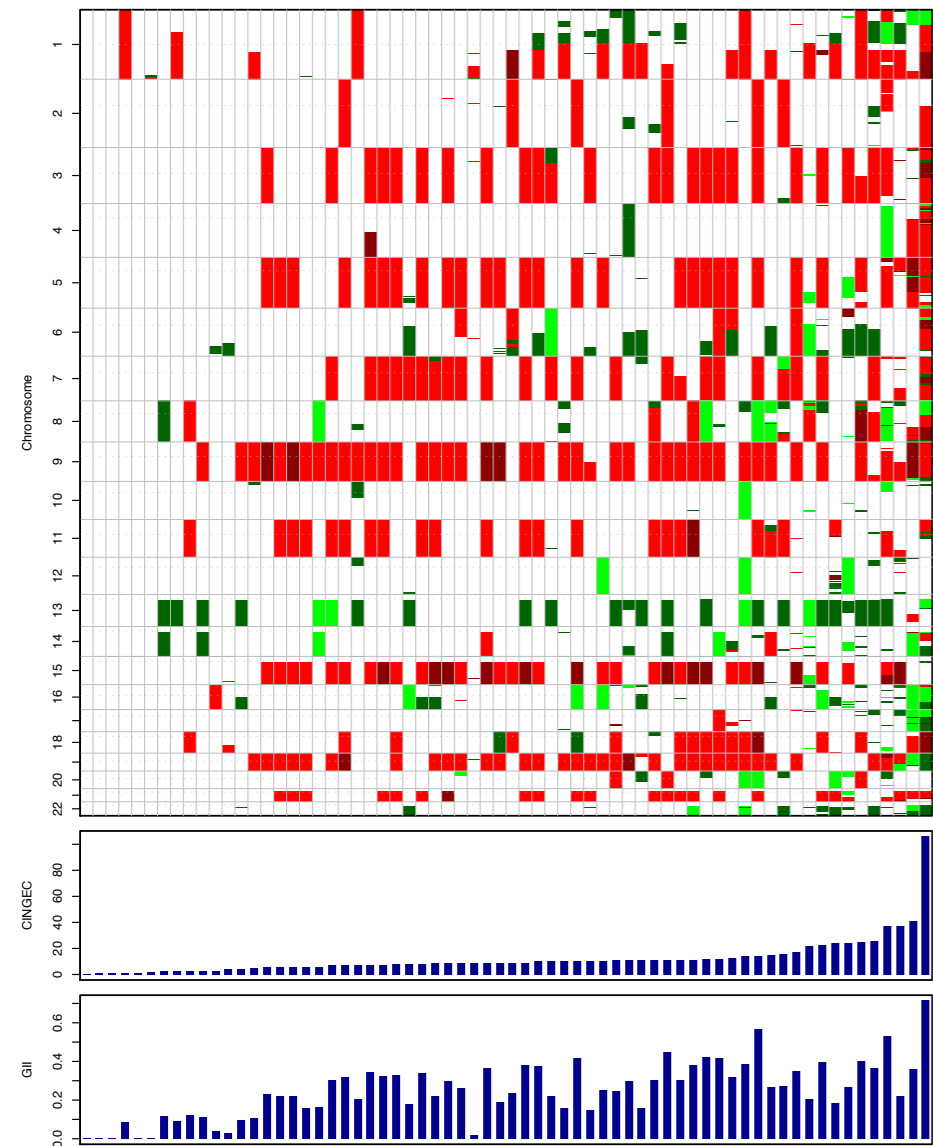

(a)

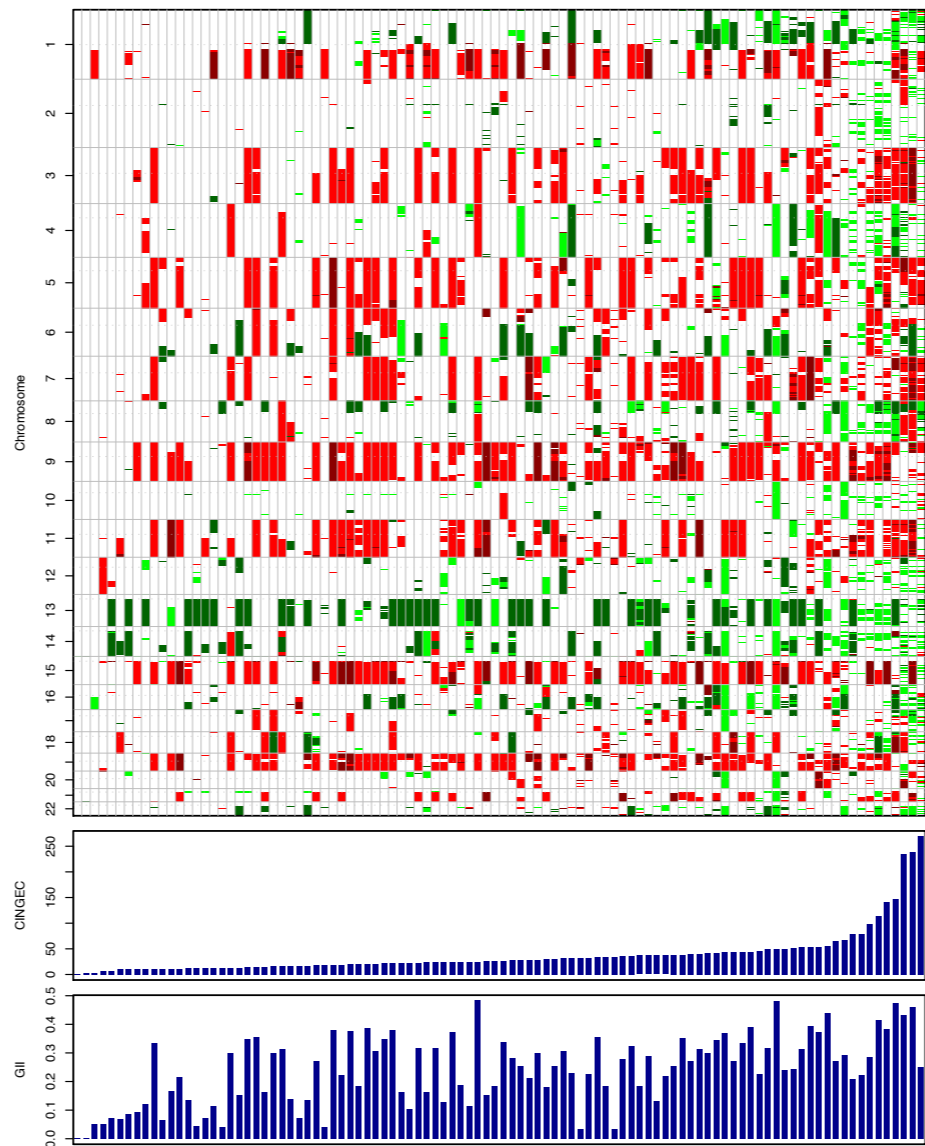

(b)

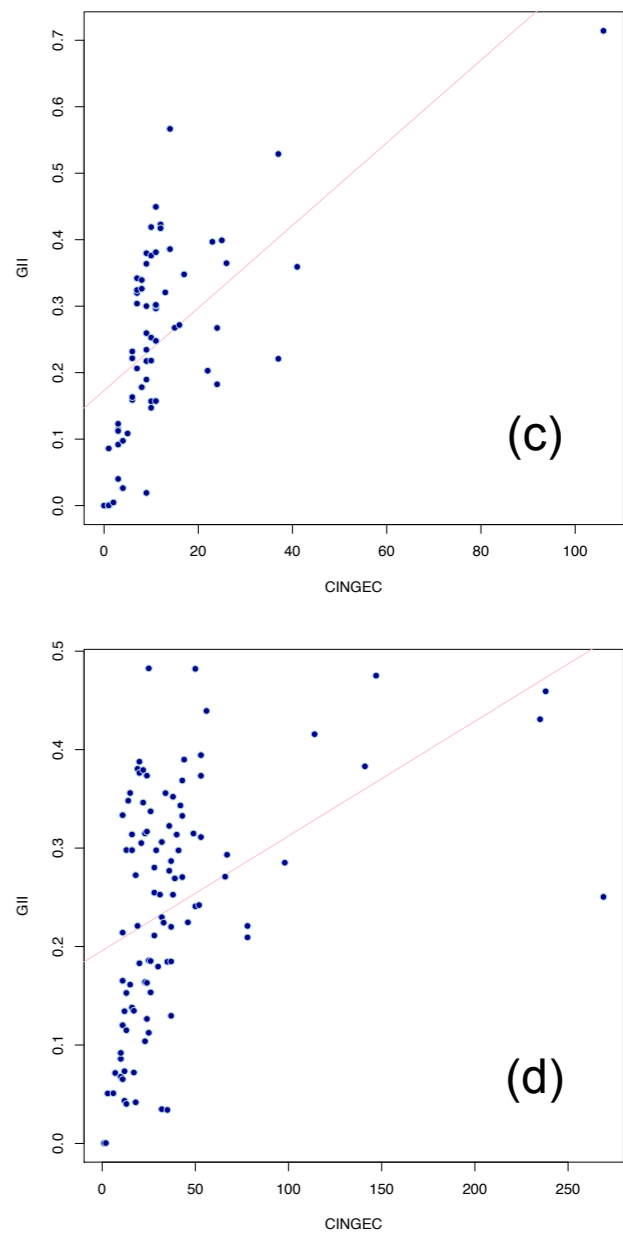

(c)

(d)

Supplement: Figure S1 — Genome wide segmentation heatmaps and scatter plots of CINGEC vs GII. Genome wide segmentation heatmaps of (a) Mayo patient primary sample aCGH data and (b) MMRC reference collection aCGH data, both in 1MB resolution. Samples are ordered according to increasing orders of CINGEC (middle panels). GII scores are displayed (bottom panels) for reference. Scatter plots between CINGEC vs GII for (c) Mayo patient data and (d) MMRC reference collection data are also shown. In heatmaps, single and 2+ copy number gains are shown in red and dark red, respectively. Likewise, single and 2 copy number losses are shown in greed and dark green, respectively. (PDF) [file pone.0066361.s001.pdf]

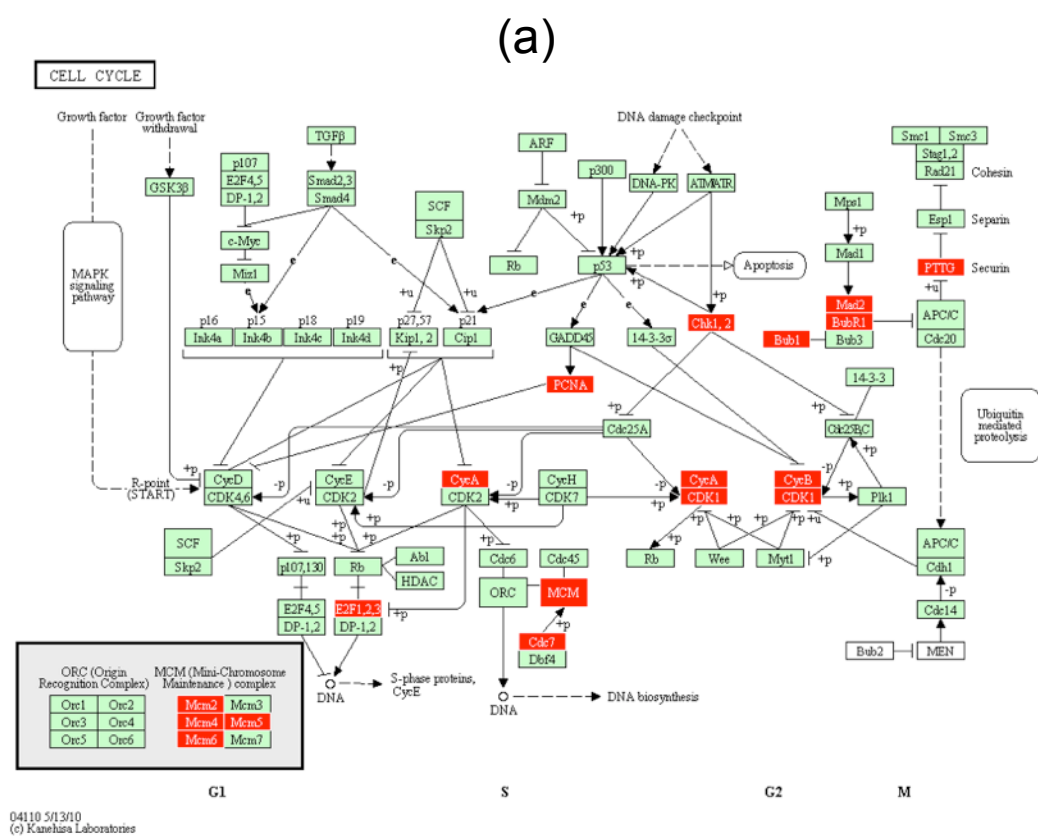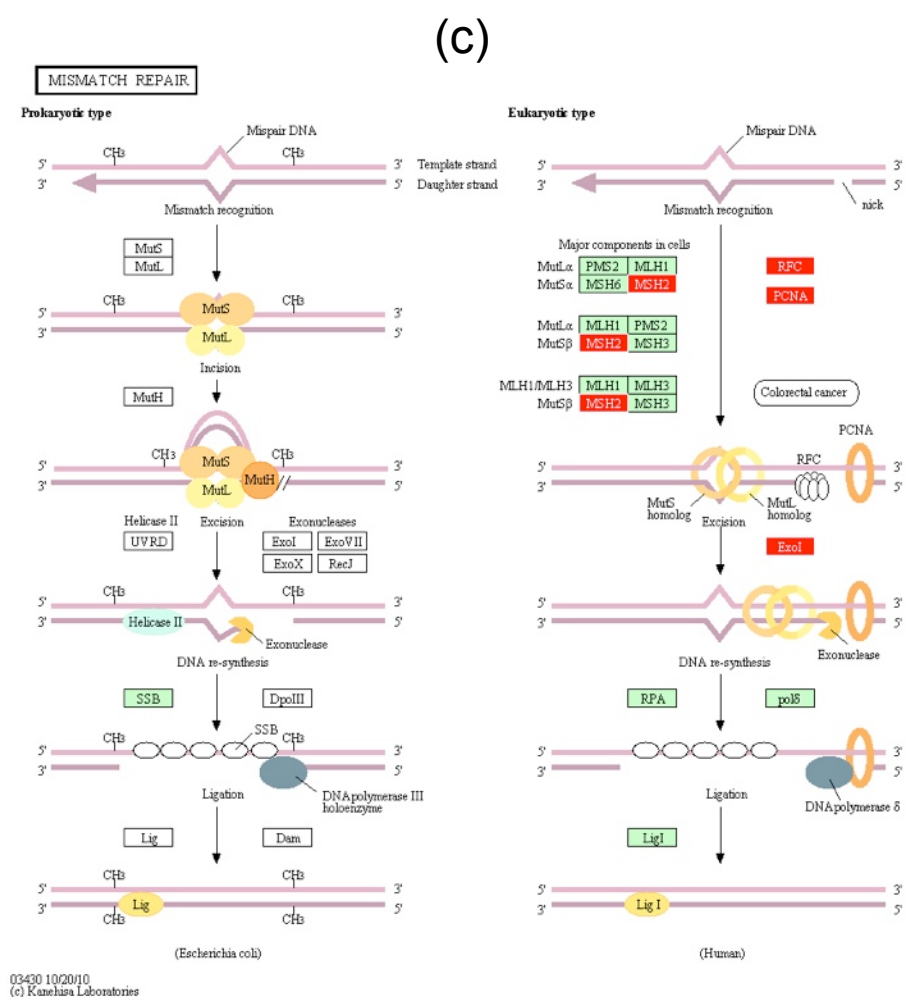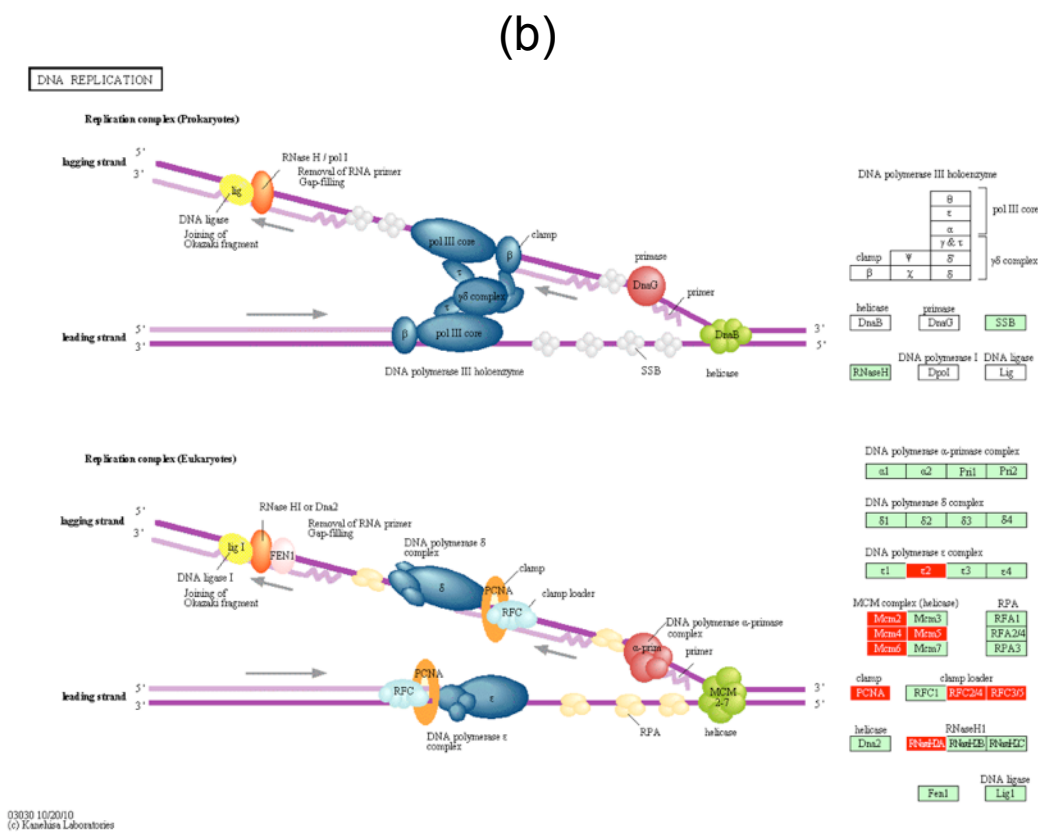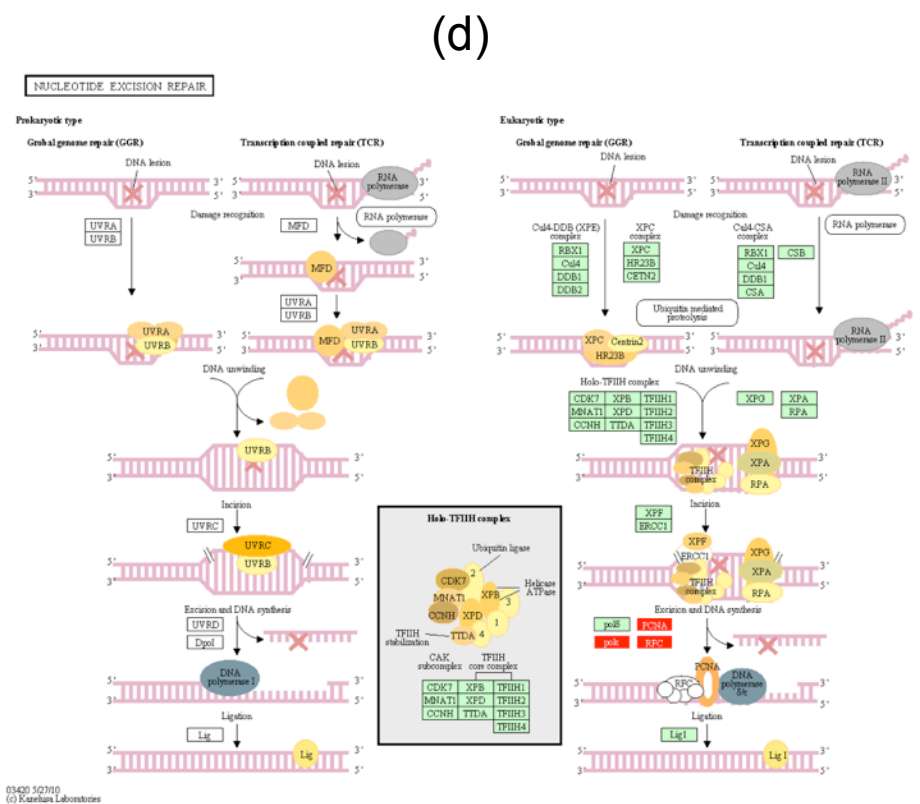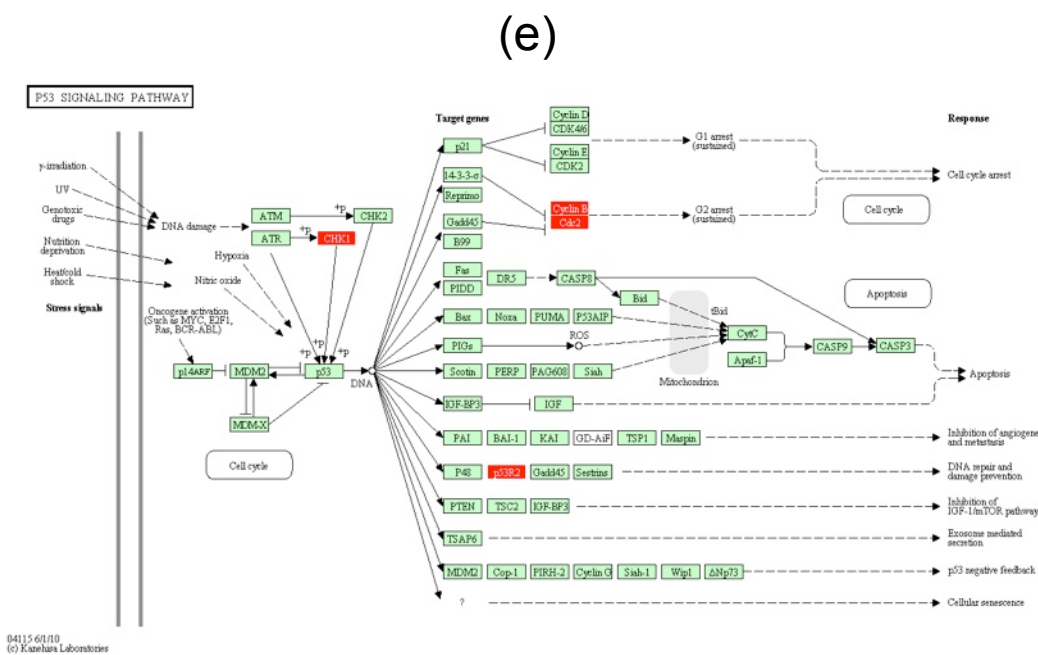

Supplement: Figure S2 — KEGG pathway diagrams for pathways proved to be statistically significant from impact factor analysis (p<0.05); (a) cell cycle (hsa04110), (b) DNA replication (hsa03030), (c) mismatch repair (hsa03430), (d) nucleotide excision repair (hsa03420), (e) p53 signaling pathway (hsa04115). Over-expressed genes are indicated as red boxes in all diagrams. (PDF) [file pone.0066361.s002.pdf]

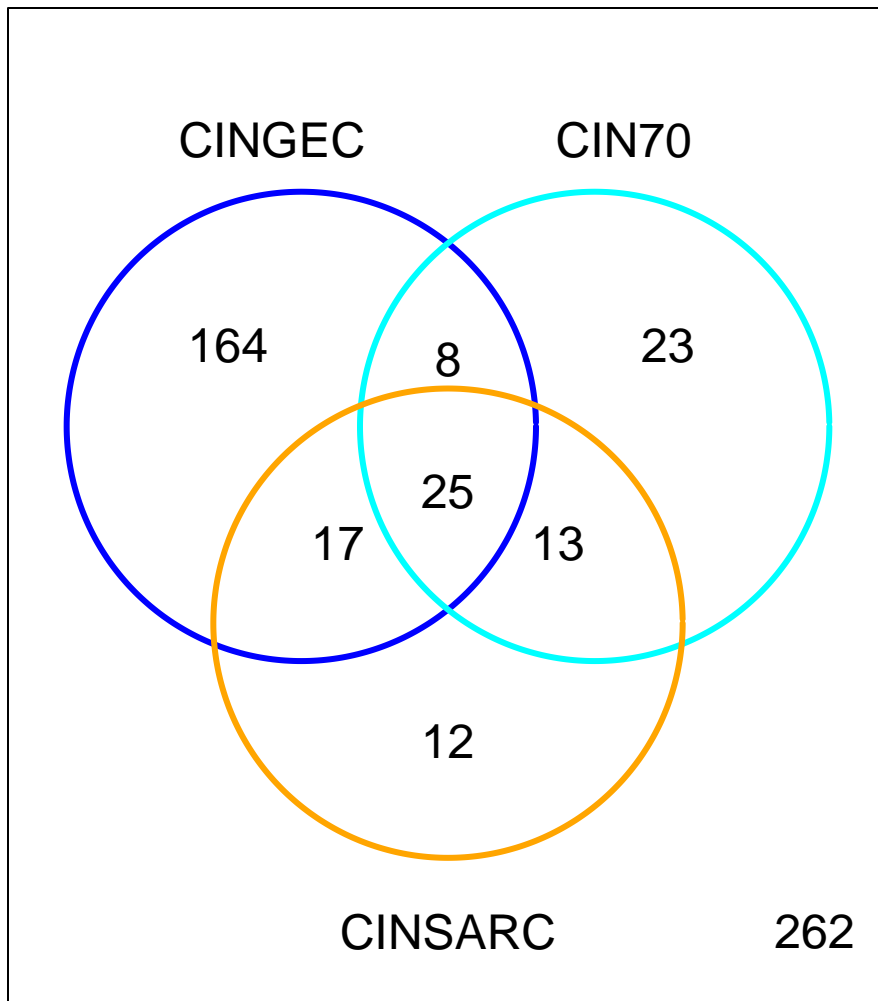

Supplement: Figure S3 — Venn diagram of intersections among probesets in 3 CIN-related gene signatures. (PDF) [file pone.0066361.s003.pdf]

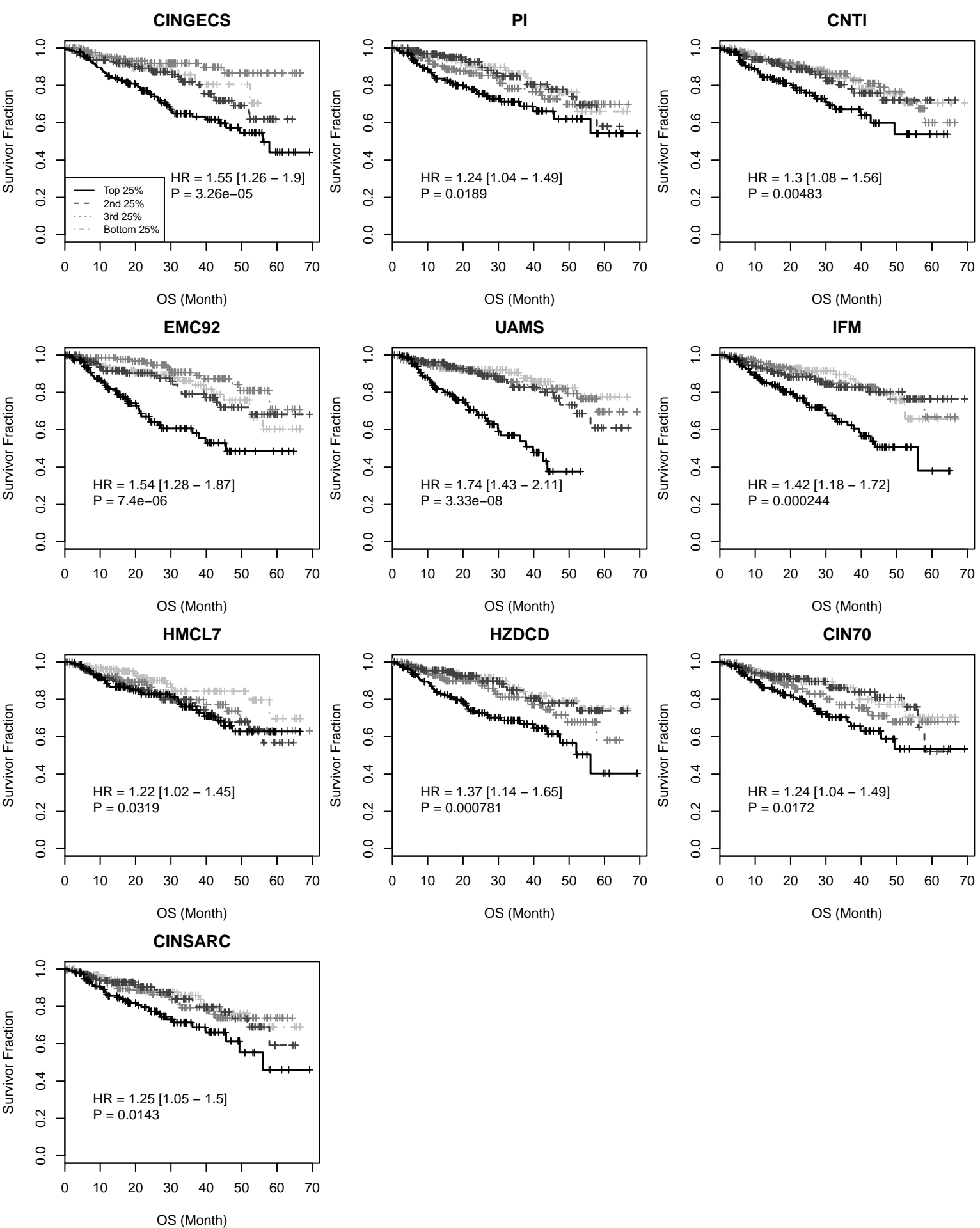

Supplement: Figure S4 — Univariate survival curves for OS in UAMS dataset among inter-quartile risk groups of gene signature indices. (PDF) [file pone.0066361.s004.pdf]

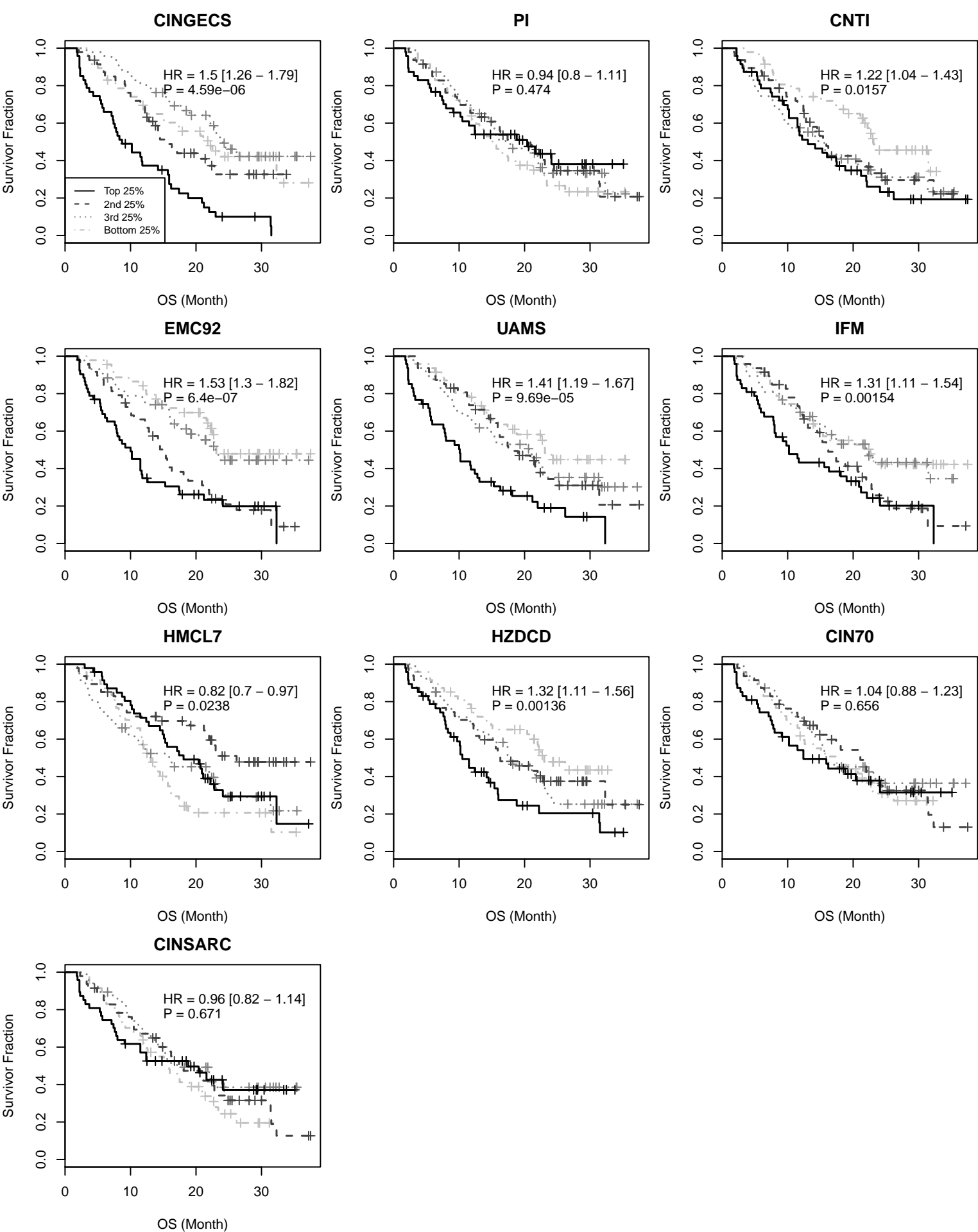

Supplement: Figure S5 — Univariate survival curves for OS in APEX dataset (bortezomib treatment cohort) among inter-quartile risk groups of gene signature indices. (PDF) [file pone.0066361.s005.pdf]

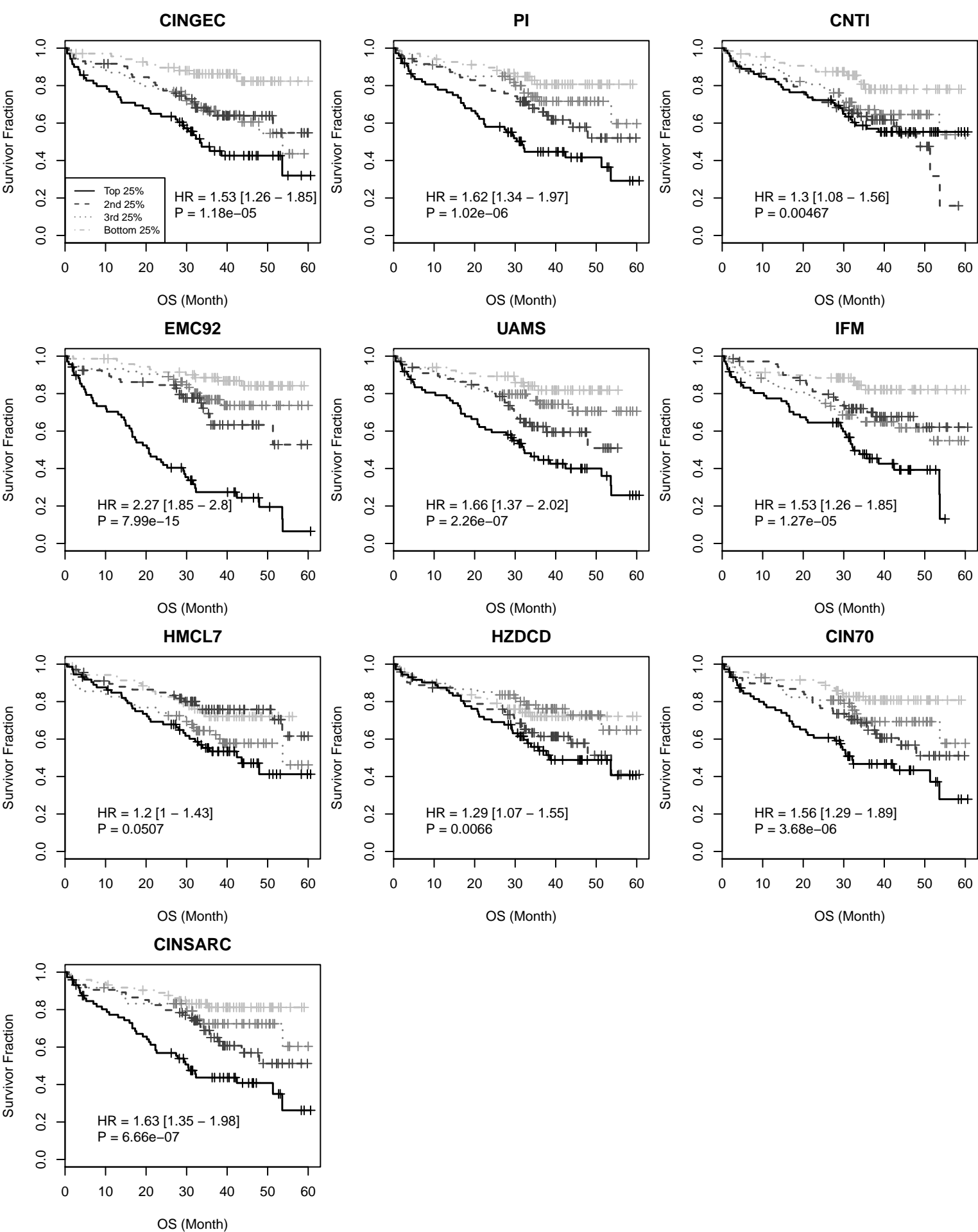

Supplement: Figure S6 — Univariate survival curves for OS in HOVON dataset among inter-quartile risk groups of gene signature indices. (PDF) [file pone.0066361.s006.pdf]
