# Supplementary material for: A Novel Measure of Chromosome Instability Can Account for Prognostic Difference in Multiple Myeloma
Source: PLoS One. 2013 Jun 20;8(6):e66361. doi: 10.1371/journal.pone.0066361 (PMC3688789; doi:10.1371/journal.pone.0066361)
Supplement: Method S1 — Supplementary Method. (DOC) [file pone.0066361.s011.doc]

# Supplementary Method

**Chromosome instability genome event count (CINGEC)**

Chromosome instability (CIN) represents the tendency for a cell to be lenient towards compromises against genome integrity. A cancer cell of more unstable genome will harbor more aberration events, and the degree of CIN of a genome can be assessed by counting the number of aberration events it harbors. Consequently, we introduce CINGEC, the *minimum* number of aberration events present in the genomic profiles, as a novel measure of CIN. Under CINGEC, cancer genomes are regarded unstable not only when they allow many numerical alterations as in aneuploid cancer genomes but also when they allow many structural alterations. In CINGEC, we consider genomic alterations directly related to copy number abnormalities that would be easily discerned from technologies like aCGH or SNP arrays. This may seem to exclude an important category of aberration events, translocation events, that can create fusion genes that are important in terms of cancer initiation in CINGEC estimation. However, evidences suggest that many of those translocations indeed carry copy number alteration events[1-3] and are readily detected by aCGH or SNP arrays. The generation of fusion genes can be understood as a consequence of opportunistic juxtaposition of DNA segments of “mistaken identity” in this sense. Obviously, translocation breakpoints without copy number alteration events can only be detected by whole genome sequencing.

Even in this restricted circumstance, we need to introduce several assumptions to make the estimation of aberration events more concrete. First, we assume gains and losses are equally probable in all genome regions regardless of their copy number status. Second, we assume aberration events can happen anywhere of genome with equal probability even for regions like centromeres and telomeres that are already known to be hotspots for genome alterations. It is already known that some structural characteristics can be associated with the appearance of genomic break points.[4-8] For instance, AT binucleotides and Alu elements are associated with the appearance of breakpoints.[4,8] Fragile sites are also known to be associated with genome abnormality.[6,7] However, the association is of purely statistical nature and it is uncertain in what degree these associations can contribute favorably or unfavorably to the appearance of breakpoints. One consequence of this assumption is that the chance to observe different events to share the same breakpoints will be very slim. For instance, if we observe a copy number gain with more than single copies, we do not assume it is an accumulation of multiple aberration events; rather, we assume it is a single event of multiple copies. Another consequence of this assumption is that the sizes of aberrant segments will be random and all aberrant segments will contribute equally to the estimation of the CINGEC index irrespective of their sizes. This is a contrasting difference to the assumption of ploidy consideration and many CIN measures that are fundamentally based on ploidy (such as the genome instability index (GII)[9] that simply assesses the fraction of aberrant regions among the whole genome) where aberration events spanning the whole chromosome or arm are considered.

0

-1

-2

2

1

(A)

(B)

(C)

(D)

# of event = 1

# of event = 1

# of event = 2

# of event = 3

Chromosomal CINGEC = 1 + 1 + 2 + 3 = 7

(A)

(B)

(C)

(D)

Copy number sequence = (0, -1, 0, 2, 0, -1, -2, -1, 0, 1, 2, 0)

Aberrant subsequences: S_(A)_ = (-1), S_(B)_ = (2), S_(C)_ = (-1, -2, -1), S_(D)_ = (1, 2)

The CINGEC algorithm starts from a copy number sequence s = (s[1], ..., s[n]), (s[i] ∈ {-p, ..., q} p, q > 0; s[i] ≠ s[i+1]) for a chromosome. Here, positive and negative values represent levels of gains and losses, respectively. Obviously, the sequence would be composed of a series of aberrant subsequences delimited by normal copy number segments. For instance, the copy number profile of an imaginary chromosome shown in the diagram above can be represented as a copy number sequence s = (0, -1, 0, 2, 0, -1, -2, -1, 0, 1, 2, 0) and it can be decomposed like s = (0) + s_(A)_ + (0) + s_(B)_ + (0) + s_(C)_ + (0) + s_(D)_ where s_(A)_ = (-1) and s_(B)_ = (2), s_(C)_ = (-1, -2, -1) and s_(D)_ = (1, 2). In CINGEC, the number of aberration events of a chromosome is estimated by the sum of aberration events from aberrant subsequences. The number of aberration events for the example chromosome would be the sum of aberration event estimates from aberrant segments s_(A)_, s_(B)_, s_(C)_ and s_(D)_. The number of aberration events for each aberrant segment increases by 1 if copy number transits into new level (s[i] ∉ {s[j] (j < i)}) (as in s_(A)_ and s_(B)_ for instance) or it transits into a previous level that is at least two steps prior to present segment (s[i] = s[m], m < i-1) (as in the purple triangle point in s_(D)_ for instance) for each chromosome. The latter criterion is based on the observation that the chance of two or more boundaries of independent aberration events coinciding with each other is very slim; rather, it is more natural to assume an intervention of another aberration event that forces different breakpoints align with each other. One final detail is that once the copy number sequence of an aberrant segment returns to some previous copy number level, all intermediate copy number levels from departing to returning events should be treated as if they are spliced out and moves to next copy number level. According to this algorithm, the aberration event estimates of aberrant segments s_(A)_ and s_(B)_ are 1, that of s_(C)_ is 2 and that for s_(D)_ is 3. In s_(C)_, the aberration event count is 2 since it increases at 0 🡲 1 and 1 🡲 2 transitions but not a 2 🡲 1 and final 1 🡲 0 transitions since they are transitions to previous levels. However, the aberration event count in s_(D)_ gains extra 1 since copy number level 2 becomes abruptly 0 (a level prior to its previous one). Consequently, the final number of aberration events for this chromosome would be 7. The final CINGEC estimate for a sample is the sum of all aberration event estimates from autosomal chromosomes to avoid confusions due to sex chromosomes.

**Determination of copy number sequence**

To be able to utilize CINGEC algorithm, we need a sequence of copy number profile that should be obtained from aCGH or SNP array data. Although this involves simple application of some thresholds to determine whether a group of consecutive aCGH or SNP array raw data are considered to be aberrant or not, actual selection of thresholds is far from trivial. In this study, we employed a simple approach. We determined the copy number level thresholds by studying the distribution of average logarithm (base 2) of ratio (logratio) between signals from cancer samples and those from reference samples in segmentation results rather than original probe-level data. Compared to an approach that examines the distribution of individual logratio values from individual probes, this approach has the advantage of trimming down the huge multiplicity of individual probe logratio values from the same copy number level segments. Coupled with the increased density of probes for recent high-throughput platforms, the problem of multiplicity and elevated signal noise is particularly pronounced for segments of normal copy number since even samples of the most distorted genomes have much more normal segments than aberrant ones. Because of this, the distribution of logratio values from normal segments is so dominant and noisy that the distinction between logratios from normal segments and those from aberrant ones is practically impossible when probe signals are used directly. However, when the distribution of average logratios from segments is examined, one can observe clear separation. Another remark is that, since CINGEC traces the history of aberration events, it is much beneficial to distinguish genome alteration status more carefully than simple gain-loss identification. Practically, this means that it is better to use more than one genome level thresholds for gains and losses, respectively, to distinguish more pronounced alterations from single copy gain/loss. If we distinguish just the gains/losses and do not pay close attention to copy number level changes within gains or losses, CINGEC estimation will be similar to CIN indices such as GII.[9]

**Reference**

1 De Gregori M, Ciccone R, Magini P, *et al.* Cryptic deletions are a common finding in ‘balanced’ reciprocal and complex chromosome rearrangements: a study of 59 patients. *J Med Genet* 2007;**44**:750–62.

2 Gajecka M, Gentles AJ, Tsai A, *et al.* Unexpected complexity at breakpoint junctions in phenotypically normal individuals and mechanisms involved in generating balanced translocations t(1;22)(p36;q13). *Genome Res* 2008;**18**:1733–42.

3 Howarth KD, Pole JCM, Beavis JC, *et al.* Large duplications at reciprocal translocation breakpoints that might be the counterpart of large deletions and could arise from stalled replication bubbles. *Genome Res* 2011;**21**:525–34.

4 Abeysinghe SS, Chuzhanova N, Krawczak M, *et al.* Translocation and gross deletion breakpoints in human inherited disease and cancer I: Nucleotide composition and recombination-associated motifs. *Hum Mutat* 2003;**22**:229–44.

5 Chuzhanova N, Abeysinghe SS, Krawczak M, *et al.* Translocation and gross deletion breakpoints in human inherited disease and cancer II: Potential involvement of repetitive sequence elements in secondary structure formation between DNA ends. *Hum Mutat* 2003;**22**:245–51.

6 Durkin SG, Glover TW. Chromosome Fragile Sites. *Annu Rev Genet* 2007;**41**:169–92.

7 Lukusa T, Fryns JP. Human chromosome fragility. *Biochim Biophys Acta* 2008;**1779**:3–16.

8 Bayani J, Selvarajah S, Maire G, *et al.* Genomic mechanisms and measurement of structural and numerical instability in cancer cells. *Semin Cancer Biol* 2007;**17**:5–18.

9 Chin SF, Teschendorff AE, Marioni JC, *et al.* High-resolution aCGH and expression profiling identifies a novel genomic subtype of ER negative breast cancer. *Genome Biol* 2007;**8**:R215.
